# The Relative Importance of Flies and Water Supply in Spreading Disease1Read at the regular meeting of the Buffalo Sanatory Club, December 14, 1898.

**Published:** 1899-04

**Authors:** M. A. Veeder

**Affiliations:** Lyons, N. Y.


### THE RELATIVE IMPORTANCE OF FLIES AND WATER SUPPLY IN SPREADING DISEASE.[1]

By M. A. VEEDER, M. D., Lyons, N. Y.

THE diseases that may be borne in water under some circumstances and by flies under others are typhoid fever, yellow fever, certain forms of dysentery, Asiatic cholera, and perhaps malarial fever, and malarial poisoning in general.

It will be observed that there are two classes of these diseases, the intestinal and the malarial. In the former the infection is a bacillus of some sort, whose presence in any locality can always be traced to some preceding contamination by excretions from a diseased bowel. In the latter the source of infection is a plasmodium, as it is termed, which lives primarily in marshy soil or stagnant water, and whose presence in that location is independent of contamination from human sources. These distinctions are to be kept in mind constantly in attempting to trace out the manner in which these diseases spread, and in adopting measures for their prevention.

---

1 Read at the regular meeting of the Buffalo Sanatory Club, December 14, 1898.

All are most emphatically camp diseases, and hence the propriety of their discussion at a meeting devoted to the subject of Camp Hygiene. There is the greater need of such discussion because the relative importance of flies and water supply in spreading disease has been greatly misunderstood, especially in the very case of camps where the danger is greatest.

Contrary to ideas that have existed heretofore, in camps and under similar conditions elsewhere, intestinal diseases are spread almost exclusively by flies, and malarial diseases by water. The exceptions to this, which are relatively unimportant, will be noted in the course of the discussion.

In the Cuban campaign last summer it was sought to prevent the spread of diseases of the intestinal class by the use of water from the purest sources or purified by boiling. It was a failure. Not until late in the summer was the agency of flies taken into the account in any practical way. Even now reports from Honolulu and Manila state that the typhoid is still epidemic among the soldiers in those localities, showing that adequate measures of prevention have not yet been perfected. Likewise, during the recent British campaign in Fashoda, which was most carefully planned, and which took place in a climate that is exceptionally dry and hygienic, there was no abatement of typhoid fever, it continuing to be the chief scourge of the army.

Nevertheless, diseases of the typhoid type can be prevented with almost absolute certainty, and malarial diseases greatly abated, in both instances by very simple measures. It is only necessary that those most concerned should think it worth while to understand the matter and act accordingly.

It is not possible to enter very much into detail in a brief summary of results such as the present. Suffice it to say that in the performance of his duties as health officer and practising physician, the writer has been able to put an end to epidemics of typhoid fever and dysentery of the exact type that prevail in the army in such manner as to warrant the belief that the problem of their prevention has been solved completely.

For example, the outbreak of malignant dysentery mentioned in my paper at the recent meeting of the American Public Health Association was encountered when at its height, there having been forty cases and ten deaths, the disease spreading rapidly from house to house in a single neighborhood. As soon as such measures were adopted as would make conveyance of the infection by flies impossible, there was not another new case, although there had been fresh ones every day for some time previously. In like manner, the past summer, a lively epidemic of typhoid fever was ended in a day by measures directed against conveyance by flies. So, too, on the malarial side of the question there has been prevention, without the aid of quinine, by measures directed against conveyance by water.

It is true that under certain circumstances the poison of intestinal diseases may find its way into water and be thus conveyed, and that the poison of malaria may be taken up and carried by mosquitoes, or flies, as the case may be. Hence it is necessary to discriminate carefully in reference to the peculiarities attending these different methods of conveyance of infection.

When flies are responsible there are little neighborhood epidemics, extending in short leaps from house to house without reference to water supply or anything else in common. But when water is at fault the disease follows its use wherever it may go, the only limitation being the resisting power of the individual using it, and such household measures of boiling and the like, as tend to destroy disease germs. Epidemics spread by flies tend to follow the direction of prevailing warm winds, as though the fly wandering out doors after contact with some source of infection had drifted with the wind, but nothing of the sort is perceptible in the case of water-borne disease. In villages and camps where shallow open closets are in use, giving access of flies to the chief sources of infection, they are the chief means of its conveyance. In cities where the underground sewers carry such material beyond the ordinary reach of flies, whose migrations are not extensive, such diseases can only become very prevalent through infection of the source of public water supply. Hence in villages and camps they are usually fly-borne and in cities water-borne. When a source of public water supply has been infected in this manner, cases of the disease will occur more or less throughout the year, provided that the germ remains active at all temperatures. When the conveyance is by flies, the prevalence of the disease will be confined to a particular season exclusively. Thus in cities typhoid fever prevails at all seasons; in villages it is an autumnal disease. When the weather is dry and sultry, flies have their best opportunity to carry the disease, or in other words under the very conditions best adapted to prevent its spread if water-borne.

With this understanding of some of the more prominent general relations of the subject it becomes possible to enter into the study of details that are of the utmost practical importance. For example, in investigating a series of epidemics confined to a single neighborhood, recurring year after year, the writer, following the lines that have been indicated, was able to secure very positive evidence, both clinical and microscopical, that burying typhoid material in the ground is no protection against flies. Such a procedure simply establishes a culture bed in which the bacilli, like plants, grow toward the surface, attracted by the warmth and other effects of the sun's rays in that location. If any prevention whatever is secured by such burial of typhoid material, without disinfection, it is very transient, especially when the weather is sultry. The application of this to the care of the sinks in the army is evident. Indeed, it is the key to the situation, so far as army conditions are concerned, the danger being greatly intensified by the utter heedlessness of many of the men. Discipline as well as accurate knowledge is required in order to make preventive measures a success in this case. Indeed, unless carefully looked after, there will be neither burial nor disinfection of such material, far too often.

Furthermore, the burial of typhoid material not only fails to prevent the immediate spread of the disease; it on the contrary actually perpetuates it in the locality from year to year. This was clearly shown in the series of epidemics referred to in the preceding paragraph, which came to an end at once when this mode of disposal of such material was abandoned and proper disinfectants were used.

The subject of disinfection in general demands revision from the point of view of the present discussion. The material employed must be suitable. If of a volatile nature, like carbolic acid, it must be remembered that on evaporation it will leave nothing behind to protect against reinfection, and consequently must be used in large quantities, and often, so that not a single particle of material containing living bacilli may escape to reinfect the entire mass. Sulphate of copper in solution, on the other hand, on evaporation is left behind diffused through the material, thus prolonging its effect, and even making it permanent, if a sufficient quantity has been employed. Earth containing sulphate of copper thus diffused, becomes a good disinfectant and may be used with confidence to cover typhoid or other infected material.

The time at which disinfection is to be performed is likewise of the utmost importance. If a case of intestinal disease is in progress, the material from the diseased bowel should not be exposed to access of flies without disinfection for a single moment, but should be received directly into sulphate of copper solution, or some adequate disinfectant. It is taking serious chances to allow even a single fly to crawl over such material, and then visit articles of food and drink. No matter how cleanly the premises, or what other precautions may be taken, if instant disinfection be neglected, the danger becomes impossible to compute. On the other hand, a very little care at the right time yields a result that is an absolute certainty and that even a child can understand.

Among large bodies of men there will always be some who are negligent, and even among those who are thoughtful, the very beginnings of an attack of sickness may pass unnoticed. Thus there is constant danger that bacilli from the diseased bowel will be planted in the sink and there find opportunity to grow and propagate. Consequently there can be no security unless all excretions are disinfected instantly if possible. Just here the point that has been made in regard to sulphate of copper in solution as a disinfectant may prove to be the key to the situation. The writer has had large experience with its use for this purpose, and has been surprised many times at the way in which even so small a quantity as a pound or two diffused through several barrels of water checks putrefaction and all signs of bacterial growth in foul drains and the like. The fact that soil can be converted into a good disinfectant by its aid is likewise important. It is portable and its color is such that there is no liability of mistake, and it likewise has the advantage of showing visibly exactly how far it has penetrated. It would seem to be the ideal disinfectant for army use, if its efficiency is as great as the writer has been led to suppose. In the absence of water it can also be used dry with good effect, but a larger quantity is required.

If, however, during the exigencies of a campaign no special means whatever of disinfection are at hand, it is nevertheless possible to do much. For instance, it is a good plan to follow the example of the Indians, who change the location of their camps often. Although burying infected material is at best a very uncertain precaution as against conveyance by flies, still it may lessen the chances somewhat for a period of some hours, more or less, according to the quantity or character of the earth employed. Hence it may be resorted to as a temporary expedient, in default of a better, when a camp is to be abandoned very soon, for example, or in the interim between the hours at which disinfectants are to be applied.

If so cumbersome an arrangement as the tank fourteen feet long and two or three feet square, containing a strong solution of crude carbolic acid, to receive the excretions, as recommended by the commission appointed to investigate the spread of diseases in the army can be had, and its use insisted upon, there is no doubt but that a vast amount of sickness will be prevented. The idea of its use is certainly based upon a correct understanding of the dangers involved, and is something new in army sanitation. Perhaps inventive genius may evolve something even better, and more readily available under all of the exigencies of warfare, now that it is known precisely what it is necessary to accomplish.

Pretty much everything that has been said thus far has had reference to the prevention of conveyance by flies, this being the greatest danger in camps. Still there may be times when the use of water that is liable to contain the bacilli of intestinal diseases cannot be avoided. It may be necessary also to use water from soil in which the plasmodium of malaria is indigenous. In either case boiling is a precaution that has the merit of absolute certainty. If it is possible to light a fire and make a little coffee or tea to drink on the spot, or to fill the canteens for subsequent use, it relieves the difficulty completely and at once.

With reference to this mode of escaping malarial disease by not touching water that has not been boiled, the writer has reason to believe that the security it affords is well-nigh absolute. It is possible that there may be some small conveyance by mosquitoes which are known to harbor the malarial parasites. There is a likelihood also that flies that have come in contact with freshly upturned marshy soil may carry the infection to some extent. But the chances in both these regards are so small that they are nothing compared with drinking the soakage from swamps fairly reeking with the poison. The deepening of wells in marshy soil may be of some assistance, especially if they are of the artesian variety, not admitting water except at the very bottom of an iron pipe. This also avoids contamination with surface filth and if such water could always be had there would be no need of other precaution. Under army conditions, however, it is better to cultivate the habit of boiling the water as a protection against diseases of the malarial type especially. There are many persons known to the writer who find it cheaper and pleasanter to do this than dose themselves with quinine while undergoing slow processes of acclimatisation so-called, on any change of residence, for it is a peculiarity of malarial infection that immunity against it in one locality does not always protect in another. Hence there is the greater need of preventive measures on the part of soldiers, who are liable to be constantly changing their location.

It is evident that much was expected in a sanitary way during the recent campaign against Spain, although it was organised in such haste. The American people do not appear to be willing to tolerate the idea that any of their boys at the front should be sick at all. At any rate if there has been suffering and death they want to know the reason why. It is a healthy symptom. But sanitation under the guns of the enemy is a hard matter, implying thorough knowledge and discipline. If anywhere this should be found, it is on the part of the American soldier who exhibited such magnificent initiative at Santiago. With the tremendous efflorescence of patriotic feeling, taking the form of sanitary inquiry in the manner that it has, the Spanish-American war of 1898 may come to be known as the war that saved more lives than it destroyed many thousandfold.

### DISCUSSION.

The President: The general subject of the camp of instruction, which I wish to remark is the only camp in which the meeting is concerned—the only one which the chair will hear discussed is now open. I shall call upon the speakers in the order in which they appear on the program.

The President then called upon G. H. Norton, Assistant City Engineer:

Mr. G. H. Norton: The question of sewers introduced by Major Babcock is one of comparative simplicity. One point in the selection of a site for such a camp would be the lay or slope of the ground, which I think should be considered to quite an extent. Judging from my experience in camps, most of them were pitched so that the headquarters were nearer the high points, the ground sloping either way toward the ends of the camp. If they were placed on a side hill, so that the slope would be toward a flank and the sewers located along the lines of the sinks, kitchens and hydrants, it would require the least possible amount of drainage, and would take advantage of the rise of the ridge to bring the flow out of either end of the camp, the men's at one end the officer's at the other. The greatest amount of flow to be cared for would be from the sinks, and baths—which seem to have been omitted from the discussion,—the kitchen slops, and from the hydrants at the company's streets. Storm water can be cared for in ditches on the surface. The water from the baths and hydrants need not necessarily be cared for in the system of sewers, but would probably be necessary to flush them.

If this is to be a permanent camp of instruction, its sewerage should be carried out with some definite plan, the disposal depending upon the surroundings of the camp. If it is to be partly of a temporary character, keeping in mind the reduction of expense as far as possible, they can be constructed so as to answer for a time of boxes, carrying them along the slopes near the surface and following closely the contour of the ground, at very small cost. The disposal by dilution, that is flowing into streams, while not an ideal one, is conceded to be the cheapest and easiest method, and without objection under certain circumstances, as reported by some of the most eminent engineers of the day, with reference to Baltimore. That is, if a large body of flowing water can be reached where the amount of dilution is sufficient and out of reach for drinking purposes. Failing this, I think chemical precipitation would be desirable. If we were to undertake to use broad irrigation or filtration, I should fear flies, of which I have some horror myself, as seen around a camp.

Dr. W. D. Greene: Of course, in supposing that we are to have a permanent camp we must pre-suppose a supply of pure water, yet we sometimes find that it is not possible to procure as pure a supply as we could wish. Possibly it is pure when the camp is first located, to be later contaminated. In point of excellence I should say that spring water would be the best we could find; the next perhaps deep well water; then water from rivers and lakes, then, lastly, shallow well water. In cases of this kind, I think I have seen it stated in papers in several instances that soldiers are, as a rule, irresponsible beings to be compared to a lot of children, and it would seem to me very desirable that these men should be instructed as to the kind of water that they should drink as they are being instructed in military tactics, that they should be taught that it is very wrong to stop in a swamp and take a drink of turbid water and they could be perhaps made to understand that drinking water of this character is prohibited. Now, it is possible that with a system of pipes carried through a camp of this character, the water may be contaminated, and if this camp is to be a permanent one, used for years for the instruction of raw recruits and making soldiers of them, it may be necessary to filter the water, which would call for a great deal of expense. The best filter for ordinary use is something akin to the Pasteur filter, but of course the supply and delivery would not be large enough. The sand filters, then, it seems to me, would be the best; they are used largely in Europe and can be constructed without enormous cost. When we consider

Mr. Gardner's contract for paving we could probably put in one for a good deal less than the usual amount and of a permanent character. Then as to the location of the water supply. I visited one camp this summer where a good many regiments were located, where I found a pump which supplied one (and I do not know but what two) was so situated that the surface drainage from these two regiments came directly down to it. That camp site was picked out, I presume, by persons who were competent, but whoever located that pump at the lowest possible point of surface drainage made a mistake; at least the regiment had a great deal of sickness. I would, therefore, say that the water supply, should it be at all suspected, should be passed through a sand filter, which will take out 99 per cent. of micro-organisms. It is conceded by every one that pure water is a necessity, and in order to get it we should have to have a filter, and such a one as would filter all the water.

MAJOR EUGENE A. SMITH : I find myself somewhat out of sympathy if the subject is to be camps of instruction. I can mention two or three which are as nearly perfect as can be; for instance, Peekskill in this State and Mount Gretna in Pennsylvania, are properly arranged for the disposal of sewerage and have good water supply. I take it the subject for discussion is camps of mobilisation, such as Camp Alger, for instance.

I have learned from the essayists this evening, points which I wish I had known better last summer, when I was in charge of four companies at Camp Alger, and thinking more of military matters than medical subjects. We should then have discussed the laying of sewers, the arrangement of a deposit plant and the excreta might have been collected, disinfected and made harmless. With the matter of the water supply, we might have profited also if we had known some of the things mentioned this evening, or had given any attention to them at all.

In regard to statistics, I am very sorry the subject was not taken up because it might have been made a very interesting line of thought.

It may be said of the letters read at the beginning of the evening, that they are very interesting. The one from General Merriam speaks of the importance of this class of camps (mobilisation), saying if there had been four of them at the beginning of the war, it might have saved many lives. He mentioned that there is great difficulty under such circumstances as we found at Camp Alger in maintaining health. I would say that it is a matter of impossibility; disease is certain to break out in more or less severe form.

To begin with, one ought to say that camps are under control of the most radical kind and hygienic errors can be placed at the door of the guilty man. The right of eminent domain in a city is represented in a camp by an autocratic power; it is under the control of one man in the way of making it absolutely perfect. He is the commanding officer, and has for his advisor the surgeon in matters of hygiene. Given a pure water supply and a proper disposal of sewerage and garbage, permanent camps should be healthy. Permanent camps are those to be occupied for two to three weeks or longer. Temporary camps are those made on the march, used from twenty-four to forty-eight hours, and they do not come under discussion this evening.

With regard to disposal of sewage and garbage in permanent camps, we have three ways of disposing of it. First, of the garbage; it can be disposed of by burning, burying, or by deposit in garbage cans or boxes and transporting it away. As for burning, the permanent camp may be near the enemy and for various reasons we could not burn it. About such a camp as at Camp Black we had large heaps of refuse which would not burn well and if we left it on the ground to dry we ran the risk of infection by flies, so that burning is not always satisfactory. Under such circumstances we had to bury the garbage, digging successive sinks and burying it as it collected. If the camp is to be in use one or two months, as Camp Black, garbage can be put in boxes and contractors can collect it in wagons from day to day, much as in cities, and certain parts can thus be disposed of. The slop element can be thrown into sinks and be disinfected. The vegetable element the farmers will carry away and use for manure. At Camp Alger it was not always done because the contract with the individual owning the land gave him the control of the disposal of the garbage, and he would not allow it to be taken away unless he were paid for the privilege. Consequently it collected in heaps and became a source of infection. Bones and the fat of meat discarded by the company cooks were very often carried away for fertilizing material or soap-making, while the cooks often got money or fresh vegetables in return. However, this was soon stopped by the man who owned the land, who wanted a pecuniary recompense himself. By burying such material the ground soon became polluted, successive sinks left pit-holes in which horses would mire; when one regiment moved away another moved near or directly on the same spots, which became a source of danger to men walking across the ground, especially after a rain. Peddlers in order to get garbage for manure would give the cook or cook's assistants liquor for the privilege of carrying it away.

As to the disposal of sewage in temporary or permanent camps, sinks constructed as mentioned here tonight near the camp are always a source of danger, even with the best of care. If distant from the camp, the men will not use them at night, especially if sick, and are likely to deposit fecal matter nearer by. It would take about all the men in the camp for sentry duty to prevent sick men and vicious, lazy men using the ground between the sinks and the camp for their deposits. If placed too near and on small areas of ground, they are infested with flies. It was common to see flies in swarms in these sinks and a short time afterward; when mess call sounded, they changed their location to the neighborhood of the cook's outfit and disputed with the men their right to the food.

The next question is that of rain. When it occurs in a camp, planned as this one, (see figure in February JOURNAL, page 495,) unless it were placed on a ridge like a saddle sloping both ways, somebody is bound to get the benefit of the water from the sinks, as you have them on both flanks, and water will flow toward either the officer's end or the men's end. The question of soakage has not been taken up to any great extent. Frequently the wells were so placed that the flow of surface water, as far as one could tell without knowing the composition of the soil thoroughly and the lay of the land, would drain toward the point where the wells were placed. It was a merciful thing that we did not stay much longer at Camp Alger, for I am very certain that had the well become infected, which it must have been in time, about one or two men out of the regiment would have escaped typhoid fever, instead of two or three hundred having it. 200 or 300 out of a regiment of 1,300 men is an enormous percentage.

In a permanent camp what can be done in the way of disposal of sewage? Under the plan, as laid down by the engineering authorities, it would seem as though some pipe system might be readily arranged and a deposit plant established at a suitable point where sewage material, particularly excreta, might be collected and disinfected. I had the opportunity to see just such a plant at the Soldiers' Home at Bath, where I was assured by Col. Shepard that they had never had but one case of typhoid fever. If the permanent camp be in the nature of a camp of instruction, there is no excuse whatever for not having a perfect water supply, closets properly trapped leading into pipes, disposing of the sewage material by flow into some stream in the neighborhood—a method much objected to by Dr. Clark and very properly—or carried to some point where it can be collected for manure after disinfection. Such camps should be prepared in time of peace by the government.

A number of other thoughts occur in this connection; for instance, in a camp of instruction where men are ignorant, they should be taught. Unfortunately in the camps with which we had experience they were not instructed and until the epidemic began we did not begin covering the dejecta, nor had we disinfectants until August, and when they did come they were very irregular as to the amount, time of delivery and the way we used them. The subject might be continued indefinitely in regard to the inexperience of cooks, cooking, the kitchens; lack of tables and consequent necessity of putting dishes on the ground and eating from them; want of store-houses and the way in which our rations were sent to us to be kept in the captain's tent for fear some one would appropriate them; and later the keeping of these rations in the supply tents, where they very soon became garbage, under the influence of heat, moisture and flies.

The President: The next subject is the presentation of the question of having some portion of the hygienic camp made impervious to moisture, to emanations from the soil, and at the same time of such material as may be kept absolutely clean, absolutely hygienic. The club is aware that this matter is a little bit novel, and we present it for your inspection with all the timidity with which the newly born ordinarily come up for inspection. We believe that the principle is sound, that to make soldiers comfortable, to keep them free from contaminating inhalations, even occasionally to keep them out of the mud in the pursuit of their regular official work, is not out of place in a camp of instruction, which the chair wishes you to get into your minds as the only camp with which we have any concern. Walks and streets will be presented by Mr. H. C. Gardner, 74th Regt. N. G. S. N. Y.

Mr. H. C. Gardner: I understand in this discussion we refer to permanent camps; the matter of paving cannot be considered in connection with the temporary camp.

To begin with I may mention two or three kinds of paving, such as asphalt, an impervious material to keep the moisture from arising from the grounds into the tents and also to run it off by way of the sewers. Then there is concrete. Asphalt has its drawbacks, so has concrete. The main trouble with asphalt is that it has a bad odor, particularly in warm weather, and in very warm weather it is somewhat plastic. It is also very hot and more expensive than concrete. Concrete absorbs less heat, consequently will radiate less and will practically last forever, if properly laid. It is very hard and rigid, entirely impervious to moisture, and if properly pitched to drain, will run all surface water away. (Speaker here referred to figure printed in February JOURNAL, page 495.) The whole area occupied by the battalion and company officers should be paved in one square and have small walks leading to it, which would be somewhat expensive. Small walks should also lead to the sinks, and floors of the tents should be paved separately. If the camp were located near a place where sand or gravel could be had for the hauling, concrete would be very much cheaper than asphalt and at the same time somewhat better. The price per foot for concrete would range all the way from 10 cents to 15 cents and would be something that would last practically forever. In smaller quantities than here represented the price would be relatively higher. In a camp such as this, containing (see figure in February JOURNAL, page 495,) 141,466 square feet in these lines, the paving would cost more than the sewering and water supply combined—more than everything else. The cost of concreting spaces where absolutely needed, *i. e.*, officers' baths and sinks, hospital quarters, officers' kitchen, company kitchens and company baths and sinks, would approximate $700.

DR. A. H. BRIGGS: The subject presented is so broad that I hardly know where to begin, but will promise, however, to take but little of your time. Whenever a body of troops is seen marching away to war, the unthinking almost invariably associate their death, should it overtake them, with bullets, bayonets and swords; but the history of all wars, with one exception, shows that this is relatively a very rare cause of death to the soldier. If we go back to the Crimean war, and I give you the statistics from memory, the Russians lost 30,000 men, or about that, from wounds received in battle. They lost 600,000 from disease. In our own war of the rebellion about 104,000 men succumbed to their wounds, and 186,000 to disease. The only war that history gives us where deaths from wounds received in battle exceeded those resulting from disease was the Franco-Prussian war. But that war was short, sharp and intense. The battles were terrible in their destructiveness; the troops were surrounded with good sanitary conditions and were constantly moving. For that reason the deaths from wounds, for the first time in history, exceeded those from disease. Now, there was not a surgeon that went into the service of the government at the beginning of the late war but was familiar with all these facts. There was not a man that did not study them, that did not resolve that if possible he would prevent this terrible loss from sickness among the troops over whom he had control. The experience we had in the war just ended was short and very instructive. I thought when I left Buffalo that memorable May afternoon that I knew something about typhoid fever—the cause of its infectiousness and its spread. I am satisfied, Mr. President, that I have very much yet to learn. There are those among us who remember the old-well period in the City of Buffalo, when we used to have little groups of infection near the wells scattered about the city. Those wells were suppressed and typhoid ceased.

You remember, too, the memorable epidemic at Plymouth, Pa., where typhoid spread so rapidly through the community and was clearly traced to the water supply. There about 1.000 out of 10,000 people were infected in one borough, a hundred of them dying of the disease, all infected from one individual through the water supply. We were prone to believe that the water supply was the main, perhaps chief, possibly the only, cause of the spread of the disease. And it became known as one of the water-borne diseases.

Now what was the condition of things we were confronted with at Camp Alger? In speaking of the water supply it can be disposed of as the Professor did the subject of snakes in Ireland, there was none. There was no water supply in the early days of the camp except what God rained down on us. Later the contractors drilled down into the dirt, to the underlying rock, apparently in the most convenient places for them to dig. Some of the water was good. That for the 65th Regiment was clear, cold and odorless, but as graphically described by Major Smith and Dr. Green, the well which supplied it took the surface drainage from the whole 1st Brigade of nearly 5,000 men. We looked upon it with distrust, fearing it would become contaminated. Permit me to say that the regiment stayed on one camp ground sixty-two days, so long that the soil became so thoroughly stale that a week after they had left, and after it had rained, as some here will remember the down-pour for several days, in spite of that I sa y the deserted ground gave off an odor that we could attribute to nothing but contamination of the soil itself. Finally there broke out in the midst (2d Army Corps) typhoid fever. It struck the regiments in a peculiar way. In some regiments it would take one company. Company E of the 65th was made up of better men in a social way, men, as a rule, of better opportunities in life and men of means, it struck that company and went through it like wildfire through punk, confining itself almost entirely to that company for a long time. It was the same with the 12th Pennsylvania. One company was infected almost to a man. Why were not the rest? They had the same food, the same water, breathed the same air, slept under the same kind of tents, but in spite of all that, the disease persisted in confining itself to one company. You could not lay it to the water supply, for all drank from the same well. The cause, or source of infection, was the problem of the day; and is the unsolved problem to this hour.

The Secretary of War has a learned board whose members are still investigating that question, made up of men of national repute, like Reed, of the Army; Vaughan, of Ann Arbor; and Shakespeare of Philadelphia. They are not satisfied, I presume, as to the cause of the spread of typhoid fever in Camp Alger today. What is the relative importance, then, of flies and water in the conveyance of typhoid fever? The paper of my friend, Dr. Veeder, is one of the most instructive I have ever listened to. It was suggested by the army board, to which I have referred, that flies might possibly be a source of infection. I think myself they were. I am thoroughly convinced that in the latter part of our own experience at Camp Alger flies were the principal source of contagion. During the last few weeks every bit of excrement was covered promptly by every man; a guard stood ready to enforce the order, and it was complied with in every regiment of the corps. We went so far as to wash the dishes of the men, to boil them, we filtered the water, but I fear we "locked the door after the horse had been stolen;" the fever was there and spread rapidly in spite of all we could do to prevent it.

The most unsanitary regiment on the ground was the 3d Virginia. Their sinks in spite of everybody would at times overflow the ground in all directions. But for some reason they had the less typhoid fever than any regiment in camp. The most sanitary regiment that came to us, and one that had all of our experience to guide and warn them was the 1st Connecticut. They came down from Maine, where they had spent the summer, in the latter part of July. They had not a sick man in the regiment. They came down in perfect health, selected a good healthy spot for their camp and availed themselves of every bit of knowledge that we had gained at such great cost, priding themselves on leaving Virginia with a clear bill of health. Before we left they had sent a whole train-load back to Connecticut sick with typhoid fever; we left on the 3d of September and on the Monday following they were preparing to send another train-load of sick men home. They had one of the most alert sanitarians I have ever known for surgeon. Now, was it due to flies or their water supply? I am unable to tell. It might have been the flies, but I think their water-supply was also infected. They were on their guard against all outside contamination. They were the only regiment in the whole corps which did not go away on a practice march, which, in my opinion, is an invention of the devil in a camp of instruction. It is a dangerous thing in an infected country like Virginia, to send men away from camp to drink in every hole they come to; and bathing anywhere they can. There are not enough officers on earth to prevent dusty, hot and thirsty men under such circumstances from violating every sanitary law.

To return to the question: we know that a water supply when contaminated is dangerous. I presume that flies may be, and I fear they were, the cause to a considerable extent of the spread of typhoid fever at Camp Alger. There is one thing I wish to say in conclusion and that is, that the Second Army Corps, should the President want soldiers next year—could go into the service a great deal more efficient than before, because they doubtless have learned many things, particularly as sanitarians and how to take care of themselves.

Dr. William Warren Potter, in closing the discussion, said: In my view there are three divisions of camps which should be discussed with reference to hygiene; the permanent camp, which for the sake of simplicity we call a camp of instruction, the camp of mobilisation, which Major Smith has so graphically described, and, finally, the bivouac. If we consider the permanent camp or camp of instruction with reference to hygiene, it ought to be a problem of easy solution. With modern knowledge of sanitation and engineering, and with the economic methods of applying the money necessary to develop it at hand, it seems to me it ought not to be difficult to create an ideal camp. But it is well to determine just how large a camp we are to construct before we can understand just how it shall be erected, or how much it shall cost. Shall it be a camp for a regiment, a brigade or a division? I believe the division is as near the unit for consideration of most questions as we can reach.

We may start out with one of the clearest propositions, and that is that the most dangerous place for the soldier is in camp; or as has been so well stated by Dr. Briggs, the dangers of the firing line are far inferior to those of the camp. With regard to the question of military economics the sanitation of the camp where the soldier is to be received for the preparation of his military life, presents a problem of the utmost importance for the government to consider. Especially is it so at the present time when we are contemplating the enlargement of the army to two or three times what its peace footing has been for some years. If we have an army of 100,000 men in contemplation, then we must raise nearly 40,000 more men. These must then be trained. You have been told by Lieut. Harris that in the regular army the recruits are drilled four months before they are sent to their regiments and that they are there drilled four to six months more as recruits before being finally distributed to their companies. Why? It is for the purpose of teaching them something which nothing but time and stern tutelage will acquire—discipline. To teach them how to take care of themselves personally, is just as much a part of the instruction that the regular army officer imparts to his men as is the manual of arms. They first make the recruit clean, and then teach him how to keep clean. But, in spite of all this, I am constrained to agree with Dr. Briggs that there is no power on earth which will prevent the thirsty soldier in general from drinking almost any kind of water when on the march; he will drink where he will if he is very thirsty. However, the provident soldier, the one who has been well trained, will provide something in his canteen like cold coffee, if he is going to make a long march, and at worst he will not drink from by-places as quickly as an inexperienced man.

It is conceded that we are to discuss on this occasion the camp of instruction and having properly located it, there then are presented questions of economics and of statistics, as well as several others of great importance. The ground, it must be confessed, has already been pretty well plowed and harrowed in regard to most of these in the papers and discussion, hence I shall touch but lightly on some of the more general features. In the camp of instruction or permanent camp, such as we have under contemplation for a division, I think it would be advisable not to permit a single well to remain within a reasonable distance of the camp. I would begin, as Dr. Briggs said was done in Buffalo a few years ago, by filling up the wells nearby and then bring the water from a distance. It should be guarded so thoroughly at its source that no man could contaminate it without risking his life. It should be piped and distributed in accordance with the plans laid down by the engineers, for this is not a problem for the doctors to determine, but one for the engineers to settle. The principal point to be remembered is that we must not only start with pure water, but we must guard it so that it shall always remain clean. It is an interesting fact, and one that I trust you will pardon me for referring to, that we are discussing this question in the very neighborhood of the place where the first knowledge of typhoid poison was developed and sent forth to the scientific world. Within the County of Erie, in 1843, a distinguished physician then residing in Buffalo, Dr. Austin Flint, discovered its source, and his investigations have become one of the most classic stories in medicine.

With a pure water supply and thorough care of the excreta, the other questions, such as paving, sewering, etc., are so plainly and definitely laid open to us through our present knowledge of sanitary engineering, that it would seem to be easily within the power of the government to establish such a place for the instruction of soldiers, as shall prevent the repetition of the unfortunate circumstances which contributed to bring us much discredit during the late war. Permit me to remark just here that I think there was one very important reason that caused much difficulty last summer, one that could not be overcome—namely, the war was too short to prevent the proper establishment and maintenance of hygienic camps constructed on the lines contemplated in this discussion. We raised 260,000 men, put them into the field, fought campaigns on land and sea, captured more men and territory than has ever been done before in the history of the world, and with less aggregate loss, considering time, number of men and results, and this notwithstanding the great difficulties we had to contend with.

There is almost no end to what might be said on this interesting general subject, but I will not further tresspass upon your valuable time, except to add simply one statistic, since I was expected to say something on the subject of statistics. Notwithstanding the fact that we have been told that typhoid fever is a preventable disease, and have been excellently instructed by our friend from Lyons, Dr. Veeder, as to the manner by which the infection is conveyed, I have lately seen statistics which I have no reason to doubt, giving the death-rate per million in civil life; and how many do you suppose died of typhoid fever? You may possibly be equally astonished, as I was, to learn that it is 48,000. So, if, in civil life, out of every 1,000,000 deaths, 48,000 die of this preventable disease we need not criticise our army friends too severely when they have a death list of a few per cent. the highest point as I remember being 4.8 per cent., reached in August.

Dr. Benedict: A question I would like to ask is, how many men of a regiment, which could be taken as a type, could be reasonably expected to develop typhoid? In other words how many were immune?

Dr. A. H. Briggs: A great many cases were put down as typhoid which were not, many being malarial cases. Statistics for the regiments are gradually being evolved, which I shall be glad to furnish later.

Dr. Miller moved a vote of thanks to those who presented papers which was carried unanimously.

Meeting adjourned.

